# Pycnogenol^®^ French maritime pine bark extract in randomized, double-blind, placebo-controlled human clinical studies

**DOI:** 10.3389/fnut.2024.1389374

**Published:** 2024-05-02

**Authors:** Franziska Weichmann, Peter Rohdewald

**Affiliations:** ^1^Horphag Research, Geneva, Switzerland; ^2^Institute of Pharmaceutical Chemistry, Westfälische Wilhelms-Universität Münster, Münster, Germany

**Keywords:** Pycnogenol^®^, pine bark extract, placebo-controlled, double-blind, antioxidant, anti-inflammatory, endothelial health, cardiovascular

## Abstract

Pycnogenol^®^ French maritime pine bark extract is a well-known and thoroughly studied patented extract from the bark of *Pinus pinaster Ait. ssp. Atlantica*. In 39 randomized double-blind, placebo-controlled (RDP) human clinical trials including 2,009 subjects, Pycnogenol^®^ French maritime pine bark extract supplementation for two weeks to six months has been shown to beneficially affect cardiovascular health, chronic venous insufficiency, cognition, joint health, skin health, eye health, women’s health, respiratory health and allergies, oral health and sports performance. The mechanisms of action that can explain the respective effects on different conditions in the human body are discussed as well. As investigated in several *in vitro*, *in vivo* and in clinical studies, Pycnogenol^®^ French maritime pine bark extract showed antioxidative effects, anti-inflammatory abilities, beneficial effects on endothelial function and reinforcing effects on the extracellular matrix. The present review aims to give a comprehensive overview of currently available “gold standard” RDP trials of Pycnogenol^®^’s benefits across various health domains compared to placebo. In addition, some of the processes on which the presented effects of Pycnogenol^®^ French maritime pine bark extract are based will be elucidated and discussed. This broad overview of RDP studies on Pycnogenol^®^ in different health domains can be used as a basis for further research on applications and mechanisms of this unique French maritime pine bark extract.

## 1 Introduction

There are many pine bark extracts on the market, from different pine tree species, from different countries and with different levels of efficacy for human health (1–4). Pycnogenol^®^ French maritime pine bark extract (Horphag Research) is the most researched pine bark extract with over 450 published studies and over 160 human clinical trials, including over 12.000 subjects (5).

The French maritime pine trees that are used for Pycnogenol^®^ grow in Les Landes de Gascogne, along the coast of southwest France (6).

Pycnogenol^®^ was found to contain mainly procyanidins, their monomers catechin and epicatechin, taxifolin as well as phenolic acids (6). The total amount of procyanidins in Pycnogenol^®^ is standardized to 70 ± 5% and thus meets the specifications for maritime pine extract, described in the United States Pharmacopeia (USP) (7). Furthermore, after ingestion of Pycnogenol^®^, taxifolin, catechin, caffeic acid, ferulic acid and metabolite 1 [δ-(3,4-dihydroxy-phenyl)-γ-valerolactone] have been detected in the blood plasma of volunteers (8).

Clinical research on Pycnogenol^®^ started over 40 years ago and various health benefits of Pycnogenol^®^ have been observed and investigated. Due to its specific composition, unique specification and standardization processes, the research results obtained from studies with Pycnogenol^®^ cannot be extrapolated to other pine bark extracts. Many of the studies are conducted following a randomized, double-blind and placebo-controlled (RDP) methodology to assess the efficacy of the extract in comparison to potential placebo effects.

**FIGURE 1:**
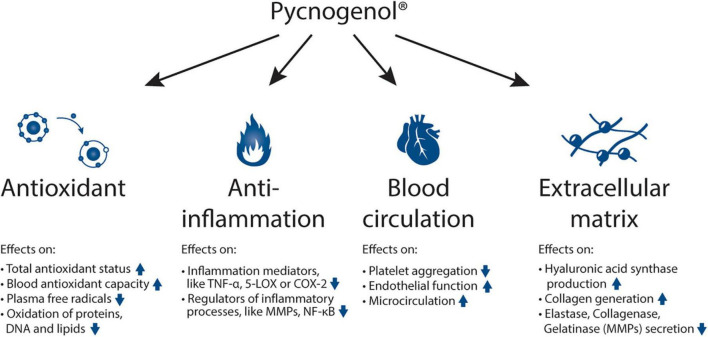
Mechanisms of action of Pycnogenol^®^: antioxidant effects, anti-inflammatory abilities, beneficial effects on blood circulation and reinforcing activities on the extracellular matrix. The respective effects of Pycnogenol^®^ that have been observed in different studies are summarized under the main effects. All these mechanisms of action of Pycnogenol^®^ are discussed in this review in more detail in the corresponding chapters. TNF-α: tumor necrosis factor alpha; 5-LOX: arachidonate 5-lipoxygenase; COX-2: cyclooxygenase-2; MMP: matrix metallopeptidases; NF-κB: nuclear factor kappa-light-chain-enhancer of activated B cells.

**FIGURE 2:**
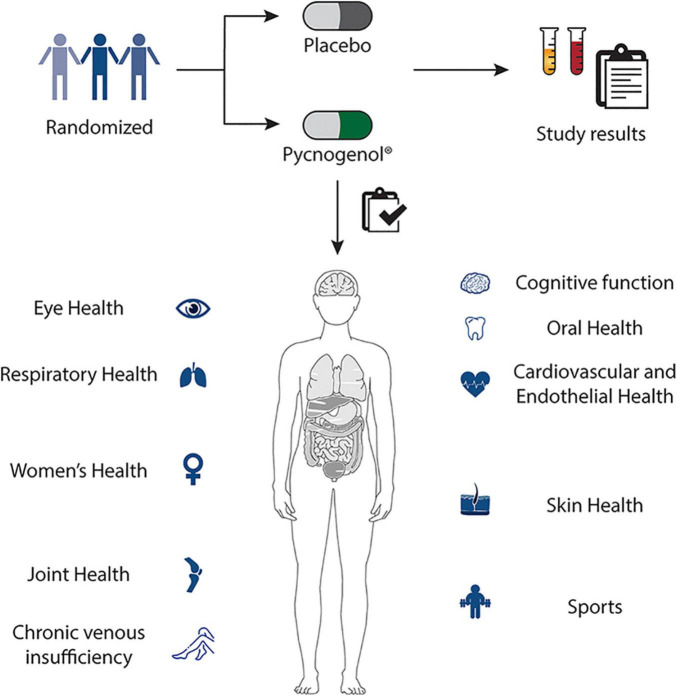
Pycnogenol^®^ supplementation has been shown in randomized, double-blind, placebo-controlled human clinical trials to beneficially affect cardiovascular health, chronic venous insufficiency, cognition, joint health, skin health, eye health, women’s health, respiratory health, oral health and sports performance. These applications of Pycnogenol^®^ are discussed in detail in the respective chapters of this review.

Pycnogenol^®^ has been shown to have four main effects being its antioxidative effects (9–14), its anti-inflammatory action (15–18), its positive impact on blood circulation (9, 19–24) and its reinforcing effects on the extracellular matrix (25, 26) (Figure 1). Mainly through these mechanisms (27), Pycnogenol^®^ supplementation has been shown in RDP human clinical trials to beneficially affect cardiovascular health (9, 11, 12, 20–22, 28–31), chronic venous insufficiency (32, 33), cognition (13, 14, 34–39), joint health (40–42), skin health (43, 44), eye health (45, 46), women’s health (12, 47, 48), respiratory health and allergies (49–51), oral health (52) and sports performance (53–56) (Figure 2).

The medicinal use of pine bark from different pine species can be traced back to Hippocrates, 400 B.C. and was applied in many parts of the world (57–60). Traditionally, pine bark has been used for their anti-inflammatory and wound healing effects (57, 61). The anti-inflammatory and antioxidant properties of Pycnogenol^®^ have since been investigated further which contributed to increased research on the versatile effects of Pycnogenol^®^ on human health, such as joint and respiratory health, cognitive function, menopause, dysmenorrhea and sports (9–18). Furthermore, Pycnogenol^®^ exerts some of its beneficial effects as it optimizes blood flow by improving endothelial function (9, 19–24). Since blood vessels run through all tissues, the effects of Pycnogenol^®^ were investigated in cardiovascular and venous health, as well as skin, hair and eye health. In addition, the composition of multiple constituents of Pycnogenol^®^, each having different benefits for human health, further explains its broad clinical application (6, 8).

For the first time, this review aims to systematically summarize and discuss these “gold standard” RDP trials comparing the efficacy of Pycnogenol^®^ to placebo and giving explanations for the different effects with the help of the respective mechanisms of action. The discussed studies were obtained from databases (PubMed, SciFinder), using the keyword “Pycnogenol” and “placebo” or “double-blind.”

## 2 Randomized, double-blind placebo-controlled human clinical studies with Pycnogenol^®^

The RDP human clinical studies conducted with Pycnogenol^®^ are summarized in Table 1, including a brief description of the study details and main findings. As the applications of Pycnogenol^®^ are so diverse, the studies are sorted by health domain and by date of publication.

**TABLE 1:** Summary of RDP human clinical studies on Pycnogenol^®^ sorted by applications and by year of publication.

References	Title	Study details	Main findings
**Cardiovascular health and endothelial health**			
Trebaticky et al. (29)	Natural polyphenols improve erectile function and lipid profile in patients suffering from erectile dysfunction	53 male subjects, 120 mg Pycnogenol^®^ per day or placebo for 3 months	Total and LDL-cholesterol levels were reduced in subjects taking Pycnogenol^®^. In diabetes type 2 patients, plasma glucose levels were decreased after Pycnogenol^®^ intake. Erectile function was improved after Pycnogenol^®^ supplementation. Placebo showed no significant effects.
Enseleit et al. (9)	Effects of Pycnogenol on endothelial function in patients with stable coronary artery disease: a double-blind, randomized, placebo-controlled, cross-over study	23 subjects, 200 mg Pycnogenol^®^ per day or placebo for 8 weeks	FMD was significantly improved by 32% in the Pycnogenol^®^ group compared to baseline and by 49% compared to placebo. Lipid peroxidation was decreased by 7% with Pycnogenol^®^.
Zibadi et al. (22)	Reduction of cardiovascular risk factors in subjects with type 2 diabetes by Pycnogenol supplementation	48 subjects, 100 mg Pycnogenol^®^ per day or placebo for 3 months	Serum endothelin 1-levels were lowered by 20% after Pycnogenol^®^ intake compared to placebo. LDL cholesterol was reduced by 12% with Pycnogenol^®^ (vs. +3% with placebo). Pycnogenol^®^ was shown to lower glycated hemoglobin by 10% in the Pycnogenol^®^ group, a significant effect compared to placebo. Fasting plasma glucose was lowered by 18.4% in type 2 diabetes subjects, taking Pycnogenol^®^ compared to placebo-controlled subjects.
Nishioka et al. (21)	Pycnogenol^®^, French maritime pine bark extract, augments endothelium-dependent vasodilation in humans	16 subjects, 180 mg Pycnogenol^®^ per day or placebo for 2 weeks	Forearm blood flow in response to acetylcholine significantly increased by up to 41% after Pycnogenol^®^ intake, while placebo had no effect. Forearm blood flow in response to an endothelium independent vasodilator was not influenced by Pycnogenol^®^ intake showing the effect of Pycnogenol^®^ is mediated by the endothelium.
Yang et al. (12)	A randomized, double-blind, placebo-controlled trial on the effect of Pycnogenol^®^ on the climacteric syndrome in peri-menopausal women	155 female subjects, 200 mg Pycnogenol^®^ per day or placebo for 6 months	LDL-cholesterol was significantly lowered, and HDL-cholesterol was significantly increased after Pycnogenol^®^ intake compared to placebo. Systolic and diastolic blood pressure were significantly reduced after Pycnogenol^®^ supplementation, compared to placebo. All climacteric symptoms improved with Pycnogenol^®^ compared to placebo.
Liu et al. (20)	Pycnogenol^®^, French maritime pine bark extract, improves endothelial function of hypertensive patients	58 subjects, 100 mg Pycnogenol^®^ per day or placebo for 3 months	Endothelin 1-levels were significantly lowered by 16% after Pycnogenol^®^ intake compared to placebo. 6-keto prostaglandin F1a-levels were increased after Pycnogenol^®^. 57% of the Pycnogenol^®^ subjects and 13% of the placebo subjects could cut their individual anti-hypertensive drug medication by half.
Liu et al. (30)	Antidiabetic effect of Pycnogenol^®^ French maritime pine bark extract in patients with diabetes type II	77 subjects, 100 mg Pycnogenol^®^ per day or placebo for 12 weeks	Plasma glucose levels of diabetes type 2 patients decreased significantly with Pycnogenol^®^, compared to placebo. Glycosylated hemoglobin and vasoconstrictive endothelin-1 in the blood were reduced and vaso-relaxant 6-keto prostaglandin f1 alpha was increased with Pycnogenol^®^ but not in placebo.
D̆uračková et al. (11)	Lipid metabolism and erectile function improvement by Pycnogenol^®^, extract from the bark of *Pinus pinaster* in patients suffering from erectile dysfunction-a pilot study	21 male subjects, 120 mg Pycnogenol^®^ per day or placebo for 3 months	Total and LDL-cholesterol were significantly reduced while HDL-cholesterol was slightly increased after Pycnogenol^®^ intake.
Hosseini et al. (28)	A randomized, double-blind, placebo-controlled, prospective, 16-week crossover study to determine the role of Pycnogenol in modifying blood pressure in mildly hypertensive patients.	11 subjects, 200 mg Pycnogenol^®^ per day or placebo for 8 weeks	Systolic blood pressure was significantly lowered after Pycnogenol^®^ supplementation by 5% compared to placebo. In subjects with the highest systolic blood pressure, the reduction was greater (−11% compared to baseline).
Wang et al. (31)	The effect of Pycnogenol^®^ on the microcirculation, platelet function and ischemic myocardium in patients with coronary artery diseases	60 subjects, 150 mg Pycnogenol^®^ per day or placebo for 4 weeks	The percentage of patients with improvement of the microcirculation at the fingertips was higher and platelet aggregation of the blood was reduced after Pycnogenol^®^ intake compared to placebo.
**Chronic venous insufficiency**			
Arcangeli (32)	Pycnogenol^®^ in chronic venous insufficiency	40 subjects, 300 mg Pycnogenol^®^ per day or placebo for 2 months	Pycnogenol^®^ supplementation reduced symptoms of CVI.
Petrassi et al. (33)	Pycnogenol^®^ in chronic venous insufficiency	20 subjects, 300 mg Pycnogenol^®^ per day or placebo for 2 months	Leg heaviness, swelling and evening edema were relieved in CVI patients after Pycnogenol^®^ intake, compared to placebo. Venous pressure was significantly decreased with Pycnogenol^®^ not with placebo.
**Cognitive function**			
Weyns et al. (37)	Clinical investigation of French maritime pine bark extract on attention-deficit hyperactivity disorder as compared to methylphenidate and placebo: Part 1: efficacy in a randomized trial	88 children, 20 or 40 mg Pycnogenol^®^ /day if < or ≥ 30 kg or 20 or 30 mg MPH /day if < or ≥ 30 kg or placebo for 10 weeks	Hyperactivity and impulsivity were significantly improved with both Pycnogenol^®^ (by 34%) and MPH (by 36%) and deteriorated with placebo, according to teacher’s rating. Inattention (according to teachers) was improved with Pycnogenol^®^ and significantly improved with MPH. The rate of adverse events was statistically significant with MPH (39%) and not with Pycnogenol^®^ (8%) and placebo (9%).
Weyns et al. (38)	Clinical investigation of French maritime pine bark extract on attention-deficit hyperactivity disorder as compared to methylphenidate and placebo: Part 2: oxidative stress and immunological modulation	88 children, 20 or 40 mg Pycnogenol^®^ /day if < or ≥ 30 kg or 20 or 30 mg MPH /day if < or ≥ 30 kg or placebo for 10 weeks	MPH intake led to loss of appetite and a significant weight loss. After Pycnogenol^®^ supplementation, children had physiologically appropriate weight gain. The orexigenic peptide, NPY was significantly reduced after MPH and insignificantly increased after Pycnogenol^®^ intake.
Donovan et al. (39)	A placebo-controlled, pseudo-randomized, crossover trial of botanical agents for gulf war illness: curcumin (*Curcuma longa*), Boswellia (*Boswellia serrata*), and French maritime pine bark (*Pinus pinaster*)	20 subjects, 400 mg Pycnogenol^®^ per day or placebo for 4 weeks	The symptoms of gulf war illness after intake of Pycnogenol^®^ were significantly reduced compared to placebo.
Ryan et al. (13)	An examination of the effects of the antioxidant Pycnogenol^®^ on cognitive performance, serum lipid profile, endocrinological and oxidative stress biomarkers in an elderly population	101 subjects, 150 mg Pycnogenol^®^ per day or placebo for 3 months	Spatial working memory and numeric quality of working memory were significantly improved with Pycnogenol^®^ compared with placebo. Lipid peroxidation products (plasma F2 isoprostane) were reduced after Pycnogenol^®^ intake compared to placebo.
Dvorakova et al. (36)	Urinary catecholamines in children with attention deficit hyperactivity disorder (ADHD): modulation by a polyphenolic extract from pine bark (Pycnogenol^®^)	61 children, 1 mg Pycnogenol^®^/kg/day or placebo for 4 weeks	The levels of catecholamines (like adrenaline, noradrenaline and dopamine) in the urine were reduced after Pycnogenol^®^ supplementation.
Chovanova et al. (14)	Effect of polyphenolic extract, Pycnogenol^®^, on the level of 8-oxoguanine in children suffering from attention deficit/hyperactivity disorder	61 children, 1 mg Pycnogenol^®^/kg/day or placebo for 4 weeks	The increased levels of 8-oxoG, as a measure of oxidized DNA in ADHD children were reduced after Pycnogenol^®^ intake compared to baseline and placebo.
Dvorakova et al. (35)	The effect of polyphenolic extract from pine bark, Pycnogenol^®^ on the level of glutathione in children suffering from attention deficit hyperactivity disorder (ADHD)	61 children, 1 mg Pycnogenol^®^/kg/day or placebo for 4 weeks	The levels of oxidized glutathione (GSSG) were significantly decreased by 22% after Pycnogenol^®^ intake, while reduced glutathione (GSH) was significantly increased by 26.8%. Placebo had no significant effect on GSSH and GSH.
Trebaticka et al. (34)	Treatment of ADHD with French maritime pine bark extract, Pycnogenol^®^	61 children, 1 mg Pycnogenol^®^/kg/day or placebo for 4 weeks	As rated by parents and teachers, hyperactivity was reduced and attention was increased after Pycnogenol^®^ intake compared to placebo.
**Joint health**			
Belcaro et al. (42)	Treatment of osteoarthritis with Pycnogenol^®^. The SVOS (San Valentino Osteo-arthrosis Study). Evaluation of signs, symptoms, physical performance and vascular aspects	156 subjects, 100 mg Pycnogenol^®^ per day or placebo for 3 months	The joint discomfort scores were reduced after Pycnogenol^®^ intake. Walking distance on a treadmill increased significantly more with Pycnogenol^®^ supplementation compared to placebo.
Cisar et al. (41)	Effect of pine bark extract (Pycnogenol^®^) on symptoms of knee osteoarthritis	100 subjects, 150 mg Pycnogenol^®^ per day or placebo for 3 months	Joint discomfort, stiffness and analgesics consumption were lowered, and physical function increased after Pycnogenol^®^ intake compared to placebo.
Farid et al. (40)	Pycnogenol supplementation reduces pain and stiffness and improves physical function in adults with knee osteoarthritis	37 subjects, 150 mg Pycnogenol^®^ per day or placebo for 3 months	Reduced joint discomfort, stiffness and need for NSAIDs with Pycnogenol^®^ supplementation compared to placebo.
**Skin health**			
Cai et al. (44)	An oral French maritime pine bark extract improves hair density in menopausal women: a randomized, placebo-controlled, double-blind intervention study	76 female subjects, 150 mg Pycnogenol^®^ per day or placebo for 6 months	Hair density of menopausal women was significantly increased after Pycnogenol^®^ supplementation compared to baseline. Scalp water loss was reduced with Pycnogenol^®^ intake compared to placebo. Resting flux of the scalp skin was improved with Pycnogenol^®^ compared to baseline.
Zhao et al. (43)	Oral Pycnogenol^®^ intake benefits the skin in urban Chinese outdoor workers: a randomized, placebo-controlled, double-blind, and crossover intervention study	78 subjects, 100 mg Pycnogenol^®^ per day or placebo for 3 months	Skin water loss was decreased while skin elasticity and skin tone regularity increased after Pycnogenol^®^ supplementation, compared to baseline and placebo.
**Eye health**			
Steigerwalt et al. (46)	Pycnogenol^®^ improves microcirculation, retinal edema, and visual acuity in early diabetic retinopathy	46 subjects, 150 mg Pycnogenol^®^ per day or placebo for 3 months	Retinal edema score and retinal thickness were reduced, and visual acuity was significantly improved after Pycnogenol^®^ intake compared to baseline and placebo.
Spadea and Balestrazzi (45)	Treatment of vascular retinopathies with Pycnogenol^®^	20 subjects, 150 mg Pycnogenol^®^ per day or placebo for 2 months	Vascular permeability of the eyes and retinal vascularization decreased, and visual acuity increased after Pycnogenol^®^ supplementation, compared to placebo.
**Women’s health**			
Kohama and Negami (47)	Effect of low-dose French maritime pine bark extract on climacteric syndrome in 170 perimenopausal women	170 female subjects, 60 mg Pycnogenol^®^ per day or placebo for 3 months	Total menopause symptom score was significantly reduced with Pycnogenol^®^ compared to placebo. Vasomotor symptoms, sleep problems and fatigue were improved with Pycnogenol^®^. Hormone levels were not changed after Pycnogenol^®^ intake, compared to baseline or placebo.
Suzuki et al. (48)	French Maritime Pine Bark Extract significantly lowers requirement of analgesic medication in dysmenorrhea–a multi-center, randomized, double-blind, placebo-controlled study	116 female subjects, 60 mg Pycnogenol^®^ per day or placebo for 2 menstrual cycles	The need for analgesic medication and the number of days on which analgesics were required was significantly reduced with Pycnogenol^®^ supplementation compared to placebo.
Yang et al. (12)	A randomized, double-blind, placebo-controlled trial on the effect of Pycnogenol^®^ on the climacteric syndrome in peri-menopausal women	155 female subjects, 200 mg Pycnogenol^®^ per day or placebo for 6 months	Menopause symptoms according to the Women’s health questionnaire were improved after Pycnogenol^®^ intake, significantly more than with placebo. The cholesterol profiles were significantly improved after Pycnogenol^®^ intake compared to placebo. Systolic and diastolic blood pressure were significantly reduced after Pycnogenol^®^ intake.
**Respiratory health and allergies**			
Wilson et al. (51)	A randomized, double-blind, placebo-controlled exploratory study to evaluate the potential of Pycnogenol^®^ for improving allergic rhinitis symptoms	39 subjects, 100 mg Pycnogenol^®^ per day or placebo for 5 to 8 weeks	Allergic rhinitis symptoms from pollen allergy, such as nasal and eye symptoms were reduced in subjects, taking Pycnogenol^®^ at least 5 weeks before pollen season. The number of subjects, requiring rescue antihistamines was reduced with Pycnogenol^®^.
Lau et al. (50)	Pycnogenol^®^ as an adjunct in the management of childhood asthma	60 children, 2 mg Pycnogenol^®^ per kg per day or placebo for 3 months	Asthma symptoms and FEV_1_ improved after Pycnogenol^®^ intake. Leukotriene levels were significantly reduced in the Pycnogenol^®^ group. The need for albuterol rescue inhalers was reduced with Pycnogenol^®^.
Hosseini et al. (49)	Pycnogenol^®^ in the management of asthma	22 subjects, 2 mg Pycnogenol^®^ per kg per day or placebo for 4 weeks	The FEV_1_ was increased after Pycnogenol^®^ intake, compared to baseline and placebo. Subjective asthma symptom rating and the level of plasma leukotrienes reduced after Pycnogenol^®^ intake compared to placebo and baseline.
**Oral health**			
Watanabe et al. (52)	Effects of French Pine Bark Extract Chewing Gum on Oral Malodor and Salivary Bacteria	21 subjects, 30 mg Pycnogenol^®^ per day in a gum or placebo gum for 4 weeks	The levels of volatile sulfur compounds in the mouth, the tongue-coating score and hydrogen sulfide-producing bacteria in saliva were reduced with a Pycnogenol^®^ gum compared to a placebo gum.
**Sports**			
Ackermann et al. (55)	The effect of an acute antioxidant supplementation compared with placebo on performance and hormonal response during a high-volume resistance training session	15 subjects, 2 ml per kg body weight of a sports drink, containing 4.8 mg Pycnogenol^®^ per 2 ml or placebo drink, single dose, 4 h before training	Muscle contractile performance and accumulated power output during lower limb hypertrophic resistance training was improved after Pycnogenol^®^ supplementation compared to placebo.
Bentley et al. (54)	Acute antioxidant supplementation improves endurance performance in trained athletes	9 subjects, 360 mg Pycnogenol^®^ in a drink or placebo drink, single dose 4 h before training	Cycling time before exhaustion was increased by 80 s after Pycnogenol^®^ drink consumption, compared to placebo subjects.
Mach et al. (56)	The effect of antioxidant supplementation on fatigue during exercise: potential role for NAD^+^(H)	13 subjects, 360 mg Pycnogenol^®^ in a drink or placebo drink, single dose, 3 h before training	The physical work capacity until fatigue during cycling training was increased with Pycnogenol^®^ compared to placebo and baseline. Serum NAD^+^ levels were increased significantly with Pycnogenol^®^ compared to placebo.
Pavlovic (53)	Improved endurance by use of antioxidants	24 subjects, 200 mg Pycnogenol^®^ per day or placebo for 30 days	Performance time on a treadmill was increased with Pycnogenol^®^ supplementation compared to placebo.

FMD, Flow-mediated dilation; LDL, Low density lipoprotein; HDL, high density lipoprotein; CVI, chronic venous insufficiency; MPH, methylphenidate hydrochloride; NPY, neuropeptide Y; 8-oxo-G, 8-oxo-7,8-dihydroguanine; ADHD, attention deficit and hyperactivity disorder; NSAIDs, nonsteroidal anti-inflammatory drugs; FEV_1_, forced expiration volume in 1 s; NAD^+^, nicotinamide adenine dinucleotide.

### 2.1 Cardiovascular and endothelial health

The effects of Pycnogenol^®^ on cardiovascular and endothelial health have been investigated more than any other health condition with ten human clinical RDP studies (9, 11, 12, 20–22, 28–31).

As the endothelium—the inner lining of blood vessels—is actively involved in many physiological functions, like controlling blood pressure, blood clotting and signaling during inflammation, endothelial function is crucial for cardiovascular health (62).

High blood pressure (hypertension) increases the risk of cardiovascular problems, such as heart attack or stroke. Vasoconstriction due to endothelial dysfunction is a common mechanism involved in hypertension related cardiovascular events (63).

In a study at the University of Zurich with 23 patients with coronary artery disease, endothelial function was assessed by measuring the flow-mediated dilatation (FMD) of the brachial artery (9). With this method, the widening of the artery in response to elevations in blood flow-induced shear stress was evaluated. 200 mg Pycnogenol^®^ per day or placebo was supplemented in a RDP cross-over study for eight weeks. With Pycnogenol^®^ supplementation, the FMD was significantly improved from 5.3 ± 2.6% to 7.0 ± 3.1% (*P* < 0.0001), compared to a slight decrease during placebo intake (5.4 ± 2.4% to 4.7 ± 2.0%; *P* = 0.051). In addition, Pycnogenol^®^’s antioxidant effects were shown by a significant decrease of 15-F_2t_-Isoprostane, a marker of lipid peroxidation from 0.71 ± 0.09 pg/ml to 0.66 ± 0.13 pg/ml, with no change in the placebo group.

The improved endothelial function with Pycnogenol^®^ leads to improved vessel relaxation, when needed, which in turn helps to normalize elevated blood pressure. These effects of Pycnogenol^®^ supplementation on blood pressure were investigated in a small RDP crossover study with eleven borderline hypertension patients (28). Pycnogenol^®^ supplementation with 200 mg a day for eight weeks significantly lowered blood systolic blood pressure from 140 to 133 mmHg compared to placebo, which was decreased only marginally. Diastolic pressure was found to be lowered as well after Pycnogenol^®^ supplementation. Interestingly, in subjects with the highest systolic blood pressure at baseline of 150 mmHg, Pycnogenol^®^ supplementation had the greatest relative normalization effect with a reduction to 134 mmHg, which translates to a reduction of 11% compared to baseline.

A study from 2007 investigated the effects of Pycnogenol^®^ on the endothelium-dependent vasodilation by measuring the forearm blood flow in response to acetylcholine, an endothelium-dependent vasodilator (21). In this study, healthy individuals took placebo or 180 mg Pycnogenol^®^ per day for two weeks following a RDP methodology. Forearm blood flow of healthy volunteers in response to acetylcholine significantly increased by up to 41% after Pycnogenol^®^ intake. Placebo had no effect. As a negative control, the forearm blood flow was measured in response to sodium nitroprusside, an endothelium independent vasodilator, which showed no change compared to baseline after Pycnogenol^®^ or placebo intake. These results show that Pycnogenol^®^’s effects on blood circulation are endothelium dependent.

In a three-month study with 58 hypertensive patients, taking the anti-hypertensive drug nifedipine (a calcium channel blocker), the plasma levels of vasoconstrictor molecule endothelin-1 were significantly lowered by 9% after one month and by 16% after three months in the group taking 100 mg Pycnogenol^®^ compared to the placebo group (20). The concentration of a vasorelaxant molecule (6-keto prostaglandin F1a as an indirect measure of thromboxane concentrations) on the other hand, increased with Pycnogenol^®^ compared to the placebo group. This is a clear indication of improved endothelial function. In the same study, the effects of Pycnogenol^®^ on blood pressure have been investigated as well. Every two weeks, the individual nifedipine dosage was adjusted so that a blood pressure below 130 mmHg was achieved. In the end of the study, 57% of the patients supplemented with Pycnogenol^®^ were able to cut their individual nifedipine medication dosage by half to keep their blood pressure in a healthy range. Only 13% of the placebo patients were able to halve their nifedipine dosage.

Another RDP study reported similar effects in 48 hypertensive patients with type II diabetes, taking an anti-hypertensive drug (an ACE inhibitor) together with 125 mg Pycnogenol^®^ daily or placebo for three months (22). At the end of the study, serum endothelin-1 levels were lowered by 17.8% in the Pycnogenol^®^ subjects compared to baseline and by 20% compared to placebo patients. In addition, 58.3% of the Pycnogenol^®^ patients were able to maintain blood pressure control with half of their hypotensive medication, which was significant compared to only 20% of subjects in the placebo group. The study found a reduction of 12% of LDL cholesterol after three months of supplementation with Pycnogenol^®^–significantly lower than the levels of the placebo group, which increased by 3% during the three months. In addition, Pycnogenol^®^ was shown to lower glycated hemoglobin by 10% in the Pycnogenol^®^ group, a significant effect compared to placebo. Also fasting plasma glucose levels decreased significantly by 16.7% compared to baseline and by 18.4% compared to placebo. A possible mechanism to understand how Pycnogenol^®^ contributes to lower glucose blood levels could be that sugar absorption was slowed down in patients supplemented with Pycnogenol^®^ by inhibition of α-glucosidase, the enzyme that disassembles starch (64).

In 21 men with moderate erectile dysfunction, supplementation with 120 mg Pycnogenol^®^ per day over a period of three months significantly decreased total cholesterol from 5.41 to 4.98 mmol/L and LDL-cholesterol from 3.44 to 2.78 mmol/L (11). HDL-cholesterol was slightly increased after three months, compared to baseline. Placebo had no effect on cholesterol levels. In addition, Pycnogenol^®^ supplementation increased the antioxidant activity of plasma determined by the FRAP (ferric reducing ability of plasma) method significantly by 15%. Using the “International Index of Erectile Function” (IIEF-5), an improvement of 33% of erectile function was determined after three months of Pycnogenol^®^ intake, while subjects taking placebo experienced a reduction of 21%. Interestingly, improvements in cholesterol levels, antioxidant effects, and erectile function were maintained even one month after termination of Pycnogenol^®^ administration.

A similar RDP study with 53 men with erectile dysfunction showed a decrease of total and LDL cholesterol by 20 and 21%, respectively, in the diabetes mellitus type 2 subgroup taking 120 mg Pycnogenol^®^ daily for three months (29). In non-diabetic subjects, taking Pycnogenol^®^, total cholesterol decreased significantly by 9% and LDL-cholesterol decreased significantly by 14%. Placebo didn’t change the cholesterol levels in any subjects. As shown before, Pycnogenol^®^ supplementation lowered glucose levels in plasma significantly by 22% in subjects with diabetes mellitus after three months. In addition, erectile function was improved by 45% in diabetes subjects and by 22% in non-diabetic patients after three months of Pycnogenol^®^ intake, assessed with the validated questionnaire International Index of Erectile Function-5 (IIEF-5).

In a RDP clinical trial with 200 peri-menopausal women, a significant decrease of LDL cholesterol by 9.9% and a significant increase of HDL cholesterol by 4.6% were observed after six months of Pycnogenol^®^ (200 mg) supplementation (12). Systolic and diastolic blood pressures were significantly lowered after Pycnogenol^®^ supplementation by 3.9 and 3.5%, respectively, while after placebo systolic and diastolic blood pressures were decreased by 1.7 and 1%, respectively.

A study with 60 subjects with coronary artery disease investigated Pycnogenol^®^’s effects on microcirculation and platelet function. After four weeks, the diameter of micro vessels at the fingertips and thus microcirculation was significantly increased in 53.8% of the patients supplemented with Pycnogenol^®^ compared to 32% of subjects of the placebo group (31). Furthermore, platelet aggregation was significantly reduced in Pycnogenol^®^ supplemented subjects compared to placebo-controlled people in the study. This was measured by using arachidonic acid and adenosine diphosphate as inducers of platelet aggregation, which were incubated with blood samples of the subjects for three minutes at 37°C.

Another RDP study showed beneficial effects of 100 mg Pycnogenol^®^ daily for twelve weeks on 77 diabetes type II patients, lowering plasma glucose levels significantly (*p* < 0.01) by 1.96 mmol/l after twelve weeks, compared to placebo (−1.11 mmol/l) (30). This effect was even more pronounced in the patient subgroup with fasting glucose levels above 10 mmol/l at baseline. Values for glycosylated hemoglobin (HbA_1_) decreased significantly in the Pycnogenol^®^ group after one month. Plasma levels of endothelin-1 were significantly lowered by over 20% with Pycnogenol^®^ while vasorelaxant 6-keto prostaglandin f1 alpha, a marker of PGI2 biosynthesis *in vivo* was significantly increased by over 10%, compared to placebo (*p* < 0.01). The researchers concluded “a remarkable recovery of endothelial function.” All patients took anti-diabetic medication in addition to Pycnogenol^®^.

### 2.2 Chronic venous insufficiency

Two RDP studies on chronic venous insufficiency have shown another interesting clinical application of Pycnogenol^®^ (32, 33) that can be mechanistically explained by its beneficial effects on endothelial function and its anti-inflammatory action (65).

An RDP study with 40 patients investigated the effects of 300 mg Pycnogenol^®^ supplementation per day for two months on subjects with chronic venous insufficiency (CVI) (32). Using a semi-quantitative scale to score the symptoms, the results showed that Pycnogenol^®^ significantly relieved swelling (−64%) and heaviness (−54%) in the legs of CVI patients compared to placebo (−7 and −3%, respectively). At the end of the study, 33% of the Pycnogenol^®^ subjects reported the complete disappearance of leg heaviness and 63% did not feel any swelling. All subjects in the placebo group still had symptoms at the end of the study.

In another RDP study on 20 patients with CVI, Pycnogenol^®^ supplementation (300 mg/day for two months) reduced venous pressure significantly by 9.8 and 8.3% (right and left legs, respectively) compared to baseline (33). In placebo subjects, venous pressure was reduced by 6.3 and 0% (right and left legs, respectively) in a non-significant way. Subjective symptoms like feeling of heaviness, swelling, and evening edema were clinically assessed using a four-item scale. After eight weeks, leg heaviness was reported to be reduced by 60.4% in the Pycnogenol^®^ group compared to a reduction of 12.6% in the placebo group. Swelling was reduced by 73.6 and 29% in the Pycnogenol^®^ and placebo group, respectively.

### 2.3 Cognition

In eight publications on RDP studies investigating the effects on cognitive function, Pycnogenol^®^ has shown to have beneficial effects in all age groups from children with attention deficit hyperactivity disorder (ADHD) to elderly people with memory-based cognitive challenges (13, 14, 34–39).

Attention deficit hyperactivity disorder is a brain hyperactivity disorder, and the most common behavioral disorder in children, with an increasing prevalence (66). A common medication for this condition is methylphenidate hydrochloride (MPH), which is associated with various adverse effects (67).

In a recently published RDP clinical trial, 88 children (aged six to twelve) with diagnosed ADHD received either Pycnogenol^®^ (20 or 40 mg/day if < or ≥ 30 kg, respectively; 20 mg/day during the first two weeks), MPH (20 or 30 mg/day if < or ≥ 30 kg, respectively; treatment started with 10 mg/ day, increasing 10 mg per week) or placebo for ten weeks (37). According to the teachers’ rating, hyperactivity and impulsivity were significantly improved by 34% with Pycnogenol^®^, by 36% with MPH and slightly deteriorated in the placebo group compared to baseline. Inattention was ameliorated as well with both remedies, the effects of MPH being significant versus baseline. The results of the parent’s ratings were similar, with both remedies showing an effect but MPH being significantly more effective on hyperactivity and inattention. However, MPH led to significantly more adverse effects in the children, including mood changes, gastrointestinal symptoms, insomnia, headache, and a feeling of tachycardia. The frequency of adverse events was 39% in the MPH group, 9% in the placebo group and 8% with Pycnogenol^®^. Additionally, MPH led to loss of appetite and significant weight loss in the children, whereas with Pycnogenol^®^, a physiologically appropriate weight gain for this age group was observed (38). Interestingly, NPY, “the hunger neuropeptide” responsible for ensuring food intake and reducing anxiety and stress was significantly reduced by 21% after MPH intake and non-significantly increased by 11% in the Pycnogenol^®^ group. The researchers concluded that with the almost complete lack of adverse effects, Pycnogenol^®^ is a good and efficacious alternative for MPH in children’s ADHD, especially in the school environment.

Another RDP clinical study could show that Pycnogenol^®^ intake relieved hyperactivity and improved attention of children with ADHD significantly compared to placebo and to baseline (34). The 61 six- to fourteen-year-old children took 1 mg/kg per day for four weeks. Using four different established questionnaires, symptoms of ADHD were assessed. According to the CAP test (child attention problem), hyperactivity and attention were ameliorated by 18.3 and 14.4%, respectively, as rated by parents and by 16.5 and 10% as rated by teachers. The difference was statistically significant compared to baseline and placebo. In the placebo group, no significant effects were observed. In addition, no side effects after Pycnogenol^®^ supplementation were reported.

Interestingly, the levels of stress hormones (catecholamines) in these ADHD-affected children were influenced by Pycnogenol^®^ supplementation (36). The concentrations of this group of hormones (including adrenaline, noradrenaline, and dopamine), that were five-times higher in children with ADHD compared to healthy children were reduced after Pycnogenol^®^ supplementation for one month. This effect of Pycnogenol^®^ partly explains its mechanism to reduce hyperactivity.

DNA damage was assessed by measuring the levels of 8-oxo-7,8-dihydroguanine (8-oxoG), representing oxidatively damaged purines (14). Oxidative DNA damage was significantly increased in all children with ADHD compared to healthy children. After intake of Pycnogenol^®^, the levels of 8-oxoG were reduced by 26.2% compared to baseline and by 35.4% compared to placebo.

In addition, Pycnogenol^®^ increased the total antioxidant status (TAS) and improved the glutathione levels, another index for antioxidant capacity in the children with ADHD (14, 35). The level of oxidized glutathione (GSSG) was significantly decreased by 22% after Pycnogenol^®^ administration, while reduced glutathione (GSH) was significantly increased by 26.8% after one month and by 36.4% after 2 months compared to baseline (35). The levels of GSSH and GSH were not significantly changed after placebo intake. These results help to further explain Pycnogenol^®^’s effects on ADHD symptoms by improving the antioxidant status.

In an RDP trial with 101 subjects, between 60 and 85 years with moderate decline of their cognitive function, the effects of 150 mg Pycnogenol^®^ per day for three months on mental performance were investigated (13). The Australian study not only followed the cognitive abilities of the subjects but also their blood profiles. Memory-based cognitive functions, more precisely the spatial working memory and quality of working memory were improved significantly by 10.9 and 8.5%, respectively, with Pycnogenol^®^ compared to placebo. Lipid peroxidation products (plasma F2 isoprostane) were reduced by 22.9% after Pycnogenol^®^ intake and by 3.7% in the placebo group. Impairment in memory skills has been found to be associated with increased age and oxidative stress (68), explaining Pycnogenol^®^’s effects on cognitive function. These findings support a beneficial effect of Pycnogenol^®^ on cognitive functions in elderly people.

In a small RDP study, the effects of Pycnogenol^®^ on gulf war illness were investigated. Gulf war illness is a chronic and multi-symptomatic disorder affecting military gulf war veterans, with symptoms like fatigue, muscle discomfort, cognitive problems, insomnia, rashes and diarrhea (69). In 20 men, taking 400 mg Pycnogenol^®^ per day for 30 days, the symptoms of gulf war illness were significantly reduced compared to placebo (39). Assessed symptoms were categorized into the six domains fatigue, pain/discomfort, skin, gastrointestinal, respiratory, and neurological/cognitive/mood, including anxiety, depressive and PTSD-related symptoms. The authors concluded that Pycnogenol^®^ may be a promising candidate to further study the effects on gulf war illness.

### 2.4 Joint health

Joint health is another essential factor for well-being. Osteoarthritis, especially in the knees, hips and hand is a very common joint problem with over half a billion people in the world living with this condition that can affect the ability to move freely (70). Osteoarthritis is a degenerative joint disorder, causing pain, swelling and stiffness (70). In three RDP studies, Pycnogenol^®^ intake has been shown to act beneficially in patients with osteoarthritis (40–42).

A pilot RDP study with 37 patients with joint cartilage damage in the knee from primary osteoarthritis showed that the intake of 150 mg Pycnogenol^®^ per day for three months led to relieved joint discomfort, with a significant reduction of 43% in self-reported discomfort, 35% in stiffness and 52% in physical function compared to baseline while placebo controls showed no significant changes (40). The difference is statistically significant compared to placebo. The need for NSAIDs (nonsteroidal anti-inflammatory drugs) or COX-2 inhibitors was high at inclusion with 23.7 ± 8.2 and 25.1 ± 9.7 pills per month for each subject in the placebo and in the Pycnogenol^®^ group, respectively. The intake of pain-relieving drugs increased in the placebo group by 3.8 pills per patient per month and was reduced in the Pycnogenol^®^ group by 15 pills per patient per month at the end of the study.

In another RDP study, 100 patients with mild to moderate osteoarthritis in the knee were supplemented with 150 mg Pycnogenol^®^ per day for three months (41). The results of this study confirmed the previous findings of decreased discomfort (by 21.4%), reduction of stiffness in the knee (by 20%) and improved physical function (by 21.7%), in comparison with placebo-controlled subjects. 38% of the included subjects, taking Pycnogenol^®^ decreased their analgesics consumption, while only 8% in the placebo group reduced the dosage.

In another RDP clinical study, 156 osteoarthritis patients, took 100 mg Pycnogenol^®^ day or placebo for three months (42). The discomfort scores decreased by 55% in Pycnogenol^®^-supplemented subjects and by 11% in placebo-controlled patients. Physical function increased by 56% in the Pycnogenol^®^ group and by 10.4% in the placebo group compared to baseline. In this study, the walking distance on a treadmill was measured as well. At the beginning of the study, all subjects could walk a mean of 65–68 m; after three months, Pycnogenol^®^ supplemented subjects were able to walk a mean of 198 m, which was significantly more than the placebo group with 88 m mean distance.

These effects of Pycnogenol^®^ on joints can be explained by previous findings, showing that regular intake of Pycnogenol^®^ leads to a potent decrease of pro-inflammatory markers like NF-κB and COX-enzymes, as well as a decline in the release of matrix metallopeptidase (MMP) enzymes, that are responsible for destroying cartilage in joints (15–18, 71).

In most cases, joint pain is due to damage to the articular cartilage. Hyaluronic acid contributes to the resistance to compression in cartilage. A small clinical study with 20 healthy volunteers demonstrated that Pycnogenol^®^ intake for four weeks increased gene expression of hyaluronic acid synthase by 44% (25). In addition, the study revealed a noticeable increase in gene expression involved in collagen *de novo* synthesis. These results are backed up by the finding of a strong increase of the concentration of Pycnogenol^®^’s metabolites and components in the synovial fluid, surrounding articular cartilage in the joints in osteoarthritis patients (71, 72). Interestingly, Pycnogenol^®^ intake in osteoarthritis patients reduced gene expression of key catabolic and inflammation markers like MMP-3, MMP-13 and IL-1β by more than 50% within knee cartilage (71). This comprehensively explains how Pycnogenol^®^ contributes to restoring health in damaged joints.

### 2.5 Skin and hair health

A study on skin health and one recently published study on hair quality have shown Pycnogenol^®^’s effects on beauty and skin health in RDP setups (43, 44).

In a RDP study, Pycnogenol^®^’s effects on the skin of 78 urban outdoor workers was investigated (43). Water loss of the skin during the hot summer season was significantly reduced by 14% with Pycnogenol^®^ supplementation for three months and only by 5% with placebo. Accordingly, skin elasticity was shown to be improved by 13% after supplementation, compared to an increase of 1% in the placebo group.

During the dry autumn season, Pycnogenol^®^ helped achieve a more even skin tone by 7.2% after six weeks and by 13.8% after twelve weeks of supplementation. These effects were statistically significant versus the placebo control. In the placebo group, subjects experienced a decrease in skin tone regularity because of pollution and sun radiation during the dry season. Skin tone regularity was assessed on the cheeks of the participants by the individual typology angle (ITA°), which is an objective classification of the skin tone in dermatology and cosmetology.

Several mechanisms behind Pycnogenol^®^’s effects on skin have been elucidated (25, 26, 73). Clinical investigations of Pycnogenol^®^ supplementation for twelve weeks in women aged 55 to 68 years found increased hyaluronic acid synthase mRNA levels by 44% and collagen type 1 mRNA levels by 40% in skin biopsies (25). These mechanisms explain the effects of Pycnogenol^®^ on skin hydration and elasticity.

Another study investigated the depigmenting action of Pycnogenol^®^ and found a significant reduction of the tyrosinase activity by 66.5%. Tyrosinase is an enzyme that activates the production of melanin, responsible for melasma in skin. In addition, Pycnogenol^®^ downregulated other pigmentation-related mediators in UV-light treated human melanocytes (73). Pycnogenol^®^’s ability to counteract skin hyperpigmentation was clinically validated in another study (26). In this clinical trial with 20 women, oral supplementation with Pycnogenol^®^ was shown to significantly lower UV-induced expression of the pigment synthesizing enzymes TRP1 by 75%, tyrosinase by 51%, MITF by 67% and MART-1 by 67%. These markers are associated with long-lasting pigmentation. From these results, it was concluded that Pycnogenol^®^ “contributes to the inhibition of pathways associated with skin hyperpigmentation” (26, 73).

A recently published RDP study identified Pycnogenol^®^ as a natural, safe and effective supplement for women who face hair thinning (44). 76 healthy menopausal women between 45 and 60 years were randomly assigned to either take 150 mg Pycnogenol^®^ per day or placebo for six months.

Remarkably, oral intake of Pycnogenol^®^ led to a significant increase of hair density of 30%, compared to baseline and of 15% compared to placebo after two months. The effects of Pycnogenol^®^ stayed on a highly improved level after six months of supplementation.

In addition, the study showed that Pycnogenol^®^ significantly reduced water loss from the skin of subjects’ scalp, compared to the placebo group. This leads to a better regulated scalp skin moisture balance for healthier hair and scalp.

The study also confirmed that Pycnogenol^®^ intake positively affects microcirculation in the skin. Using photoplethysmography, Pycnogenol^®^ was found to decrease resting flux in the scalp by 21.7% after two, and by 43.5% after six months. In the placebo subjects, the observed decrease was only 5.1 and 20.5%, respectively. This effect of Pycnogenol^®^ leads to a better supply of nutrients and oxygen to hair follicles. In several previously published studies, Pycnogenol^®^ was shown to improve circulation in small blood vessels in the body, like the very fine micro vessels in the fingertips or in the retinal capillaries of the eye (31, 46). In addition to an improvement of microcirculation, two other mechanisms of action explain Pycnogenol^®^’s efficacy for hair health and beauty. Pycnogenol^®^’s anti-inflammatory (15–18) and antioxidant activities (9–14) contribute to protecting hair follicles by capturing free radicals, generated either by stress, sun rays, pollution, or inflammation.

### 2.6 Eye health

Owing to its effects on microcirculation, inflammation and oxidation, Pycnogenol^®^ was found to have positive effects on eye health (45, 46). As the retina is the tissue with the highest metabolic rate in the body, it is particularly susceptible to oxidative stress. The eye tissue is particularly exposed to UV light which generates reactive oxygen species in cells. Furthermore, metabolic conditions like diabetes involve a pathological oxidative stress, which can lead to diabetic retinopathy (74).

Pycnogenol^®^ showed effects to stop further progression of retinopathy and ameliorate the eyesight of diabetics by stabilizing and sealing leaky capillaries of the retina, stopping further outflow of blood (45, 46).

In an RDP study, Pycnogenol^®^ was shown to reduce capillary leaking in the eyes (45). Using fluorescein angiography as a measurement tool, vascular permeability was found to have decreased by 16 and 23% for left and right eye, respectively, compared to baseline. Retinal vascularization and the presence of macular edema was assessed by ophthalmoscopy, determining the severity of retinal damage. After Pycnogenol^®^ supplementation for two months, the ophthalmoscopy score significantly improved by 9% in the left eyes and by 17% in the right eyes compared to baseline, whereas there was no change observed in the placebo group. Furthermore, Pycnogenol^®^ supplementation led to increased visual acuity by 5.7% (left eye) and 7% (right eye). The improvement was statistically significant compared to placebo. Whereas, with placebo treatment, retinopathy continued to progress as the visual acuity was reduced by 3.2% (left) and 7.5% (right), assessed by the Snellen Chart.

Another study showed that Pycnogenol^®^ reduced retinal edema and improved visual acuity in early diabetic retinopathy (46). The score assessing retinal edema was significantly reduced by 30% in mild edema cases and by 35% in moderate edema cases compared to placebo patients after three months of Pycnogenol^®^ supplementation. The retinal thickness, assessed by high resolution ultrasound, significantly decreased by 11% in mild cases and by 25% in moderate cases in Pycnogenol^®^ subjects. The most significant outcome observed in this study was the improvement of the visual acuity by 21% in Pycnogenol^®^ patients compared to baseline and placebo patients, which was assessed with the Snellen chart. Furthermore, the retinal blood flow was improved by around 30% after Pycnogenol^®^ supplementation.

Pycnogenol^®^’s effects on endothelial function (9) might explain the observed effects on perfusion of the retinal tissue and the resulting restoration of vision loss in diabetic retinopathy patients.

### 2.7 Women’s health

Investigating the efficacy of Pycnogenol^®^ on the health of women, two RDP studies on menopausal symptoms (12, 47) and one on menstrual discomfort (48) have been performed.

An RDP study with 155 peri-menopausal women found the menopause symptoms assessed by the Women’s Health Questionnaire (WHQ) to be significantly improved after six month of Pycnogenol^®^ supplementation, as compared to placebo controls (12). The symptoms on the WHQ include somatic (tiredness, headache) and vasomotor problems (hot flashes, sweating), depressed mood, memory and concentration issues, attractiveness, anxiety, sexual behavior (including vaginal dryness), sleep, and menstrual problems. Already after one month, all symptoms of the Pycnogenol^®^ supplemented subjects were significantly improved compared to enrollment and most items significantly compared to placebo. In the placebo group, several symptoms also improved significantly compared to baseline after one month, however, after six months, only memory/concentration remained significantly increased compared to baseline. Interestingly, systolic and diastolic blood pressure, as well as LDL-cholesterol levels were found to be significantly reduced after 6 months of Pycnogenol^®^ supplementation, compared to placebo. The increase of total antioxidant status, measured as Trolox equivalent antioxidant capacity, after Pycnogenol^®^ intake was highly significant compared to baseline and placebo. From these results, the authors attributed a positive, protective role to Pycnogenol^®^ on the vascular health of peri-menopausal women, which also “clearly reduced the frequency as well as the severity of climacteric symptoms” (12).

Significant efficacy of a low dosage of Pycnogenol^®^ (60 mg daily) on climacteric symptoms could be shown in an RDP investigation with 170 women (47). In this study, the total menopause symptom score of the women was reduced by 17% compared to placebo control after three months. Vasomotor symptoms (including hot flashes), sleep problems and fatigue were significantly improved in the Pycnogenol^®^ group compared to placebo. After three months, no significant effects on blood pressure or cholesterol levels were detected but positive trends were observed. In this study, blood plasma levels of different sexual hormones were investigated as well. Interestingly, after Pycnogenol^®^ intake, none of the hormone levels showed significant changes compared to baseline or placebo, suggesting non-hormonal mechanisms of Pycnogenol^®^ on climacteric symptoms.

The results from these studies demonstrated interesting efficacy of Pycnogenol^®^ supplementation in addressing various climacteric symptoms. The underlying mechanism has not been investigated fully yet, however, Pycnogenol^®^’s validated effects on blood circulation and endothelial health are good explanations for its beneficial effects on symptoms like hot flashes and sexual behavior.

An RDP multicenter study from 2008 showed that Pycnogenol^®^ supplementation significantly reduced abdominal discomfort in women with dysmenorrhea (48). The women took Pycnogenol^®^ for two menstrual cycles, during which they needed significantly less analgesic medication than in the registration period of two months before supplementation (−46%) and less than placebo controls (−28%). Interestingly, after discontinuing Pycnogenol^®^ and placebo intake in the fifth cycle of the study, subjects of the Pycnogenol^®^ group reported a continuation of the trend, as their need for analgesics was even more reduced (−50% compared to baseline), while women of the placebo group experienced a rapid return to analgesics. The number of days on which analgesics were required was significantly reduced with Pycnogenol^®^ from 2.1 to 1.3 days and changed less in the placebo group, from 1.9 to 1.7 days.

Inflammatory processes were found to be a key mechanism in dysmenorrhea (75). During menstruation, the tissue lining of the uterine cavity is replaced, leading to wound healing and inflammation. As mentioned before, Pycnogenol^®^ was shown to have potent anti-inflammatory activities in several studies (15–18).

### 2.8 Respiratory health and allergies

Chronic respiratory diseases, such as chronic obstructive pulmonary disease (COPD), asthma and pulmonary hypertension are among the five disease areas with the highest global mortality and morbidity rates (76). With almost 300 million people affected, asthma is one of the most common respiratory diseases worldwide (77). Asthma is a chronic inflammatory disease that can be triggered by allergies, by medication such as aspirin or it can arise within the body through yet unclear molecular mechanisms (78). Recurrent episodes of coughing, shortness of breath, wheezing and chest tightness are typical symptoms for asthma (79).

Supplementation with Pycnogenol^®^ has been shown to have several beneficial effects on asthma and allergic rhinitis symptoms (49–51). Especially Pycnogenol^®^’s anti-inflammatory effects are suggested to be responsible for its anti-allergic and anti-asthma efficacy.

In an RDP cross-over study, the effects of Pycnogenol^®^ supplementation for four weeks on 22 chronic asthma patients were investigated (49). The patients’ lung function was assessed by analysis of the “forced expiration volume in one second” (FEV_1_), representing the percentage of lung volume exhaled in a second. In asthmatics, the FEV_1_ is generally reduced as their breathing is restricted. After Pycnogenol^®^ supplementation, the all-adult patients could exhale 70% of their lung volume as compared to 59% at trial start and 63% in response to placebo. Additionally, the study participants rated their asthma symptoms regarding severity. Four weeks of Pycnogenol^®^ intake led to a reduction of symptom severity by 20%, whereas it was reduced by 4.5% in placebo subjects. Furthermore, Pycnogenol^®^ supplementation significantly reduced pro-inflammatory mediators (leukotrienes) in the blood of patients, as compared to both baseline values and placebo.

Most asthmatics develop the disease already during childhood, often before the age of five years. An RDP study investigated the effects of Pycnogenol^®^ on 60 children aged six to eighteen years with mild to moderate asthma (50). The study showed that the breathing capacity improved significantly already after one month of Pycnogenol^®^ supplementation, as assessed by measuring FEV_1_. The severity of asthma symptoms as well as leukotriene levels in the urine decreased drastically after one month of Pycnogenol^®^ intake and further decreased significantly throughout the trial period. The treatment with placebo had no significant effect on leukotriene levels or asthma symptoms. Another interesting outcome of the study is the reduced necessity of using albuterol rescue inhalers as severe asthma attacks appeared less frequently. After one month, eight out of thirty children taking Pycnogenol^®^ didn’t require rescue inhalers anymore and eighteen children were completely off the inhaler after the three-month supplementation with Pycnogenol^®^.

In an RDP trial, researchers found that Pycnogenol^®^ supplementation improved allergic rhinitis symptoms from birch and other pollen allergy when the supplementation started at least five weeks before the pollen season (51). Eye symptoms decreased by 35% and nasal symptoms by 20.5% compared to placebo. The number of patients requiring rescue antihistamines was 26% lower in the Pycnogenol^®^ group than in the placebo group.

### 2.9 Oral health

A study on oral health, specifically on persistent malodor (halitosis) was conducted with Pycnogenol^®^ (52). The 21 healthy participants used either two placebo gums or two gums with 2.5 mg Pycnogenol^®^ each, six times daily for 15 min each. After two weeks of Pycnogenol^®^ gum usage, the levels of volatile sulfur compounds (hydrogen sulfide, methyl mercaptan and dimethyl sulfide) were significantly reduced compared to baseline and after four weeks, these levels were reduced significantly compared to placebo. In addition, the Pycnogenol^®^ group had a lowered tongue-coating score and significantly reduced hydrogen sulfide-producing bacteria in saliva after four weeks. The mechanism behind these effects has been suggested to be the previously observed bacteriostatic properties of Pycnogenol^®^ (80).

### 2.10 Sports

In four RDP investigations, Pycnogenol^®^ supplementation was shown to enhance sport endurance and performance (53–56).

An RDP crossover study found that the endurance time of a group of recreational athletes was significantly increased after Pycnogenol^®^ supplementation (53). For the measurements, the subjects performed on a treadmill with individual setting adjusted to 85% of a person’s maximal oxygen consumption. The 20 to 35 years old participants received 200 mg Pycnogenol^®^ per day or placebo for 30 days, then switched to the other group for another 30 days. Endurance was evaluated by measuring the running time in seconds on the treadmill after 30 and after 60 days. The composite performance time of the Pycnogenol^®^ groups increased significantly by 23.2% compared to baseline and by 7.1% compared to placebo. The time of placebo subjects was increased by 15% compared to baseline.

A small RDP study with nine trained 25 to 45 year old cyclists examined the effects of a single dose of a fitness drink with Pycnogenol^®^ as the only active ingredient on performance (54). The placebo drink consisted of the same ingredients (pineapple pulp, molasses, sodium chloride, flavor, steviol glycosides, sodium benzoate, potassium sorbate), except Pycnogenol^®^. The study protocol included two separate sets of training of five min cycling at 50% peak power output (PPO), eight min at 70% PPO and cycling until fatigue at 95% PPO. The Pycnogenol^®^ or placebo drink was consumed four hours before the training. On average, the Pycnogenol^®^ supplemented cyclists rode 80 s longer until they reached exhaustion compared to subjects, taking a placebo drink. In addition, considerable evidence was given for positive effects of Pycnogenol^®^ intake on performance related variables like the rating of perceived exertion, heart rate and blood lactate concentration at 70% PPO.

In a similar RDP study, 15 resistance trained subjects in their twenties took a single dose of the same fitness drink, containing Pycnogenol^®^ as the active ingredient (55). The participants consumed the placebo or Pycnogenol^®^ drink four hours prior to a lower limb hypertrophic resistance training, consisting of six sets of 70% of the individual’s repetition maximum of back squats. This exercise was repeated at a second visit. The researchers found improved muscle contractile performance after the Pycnogenol^®^ drink supplementation during resistance training compared to a placebo drink. Accumulated power output after Pycnogenol^®^ intake was significantly higher by 4% compared to placebo controls. Over the course of the six sets, power and velocity during the resistance training sessions was stable after Pycnogenol^®^ intake but was reduced significantly with each set in the placebo group.

Another RDP study with six trained and seven untrained subjects investigated NAD^+^/NADH levels after continuous progressive exercise and after having taken the fitness drink with Pycnogenol^®^ or placebo (56). The training setup was comparable to the cycling training in the study of Bentley et al. (54). The physical work capacity until fatigue was increased by 17% in the Pycnogenol^®^ group compared to placebo. In addition, serum NAD^+^ levels were increased significantly with the Pycnogenol^®^ drink compared to placebo drink in trained and untrained subjects.

These results confirm the antioxidant properties of Pycnogenol^®^ and its protection against the high post-exercise oxidative stress.

There are several underlying mechanisms of Pycnogenol^®^ to explain these effects. During exercise, heart rate, blood flow and ventilation rapidly accelerate, and to do so, the cardio-pulmonary system adjusts to allow more nutrients, energy and oxygen to muscle tissues and avoid anaerobic build-up of lactic acid. Sufficient muscle oxygenation is facilitated by relaxed blood vessels and improved blood flow, which warrants aerobic energy generation. This increase in oxygen supply translates into acute oxidative stress due to higher production of free radicals in blood and muscles (81). As mentioned before, Pycnogenol^®^ was shown to have potent antioxidant efficacy and protects lipids, DNA and proteins from oxidation within the body in addition to its ability to increase blood antioxidant capacity (9–14).

Another property of Pycnogenol^®^ explaining its effect of increased performance is the improved tissue perfusion by enhancing microcirculation (31) and its effects on endothelial function (9, 19–24) contributing to improved blood flow by relaxing blood vessels.

## 3 Discussion

Pycnogenol^®^’s unique composition of bioactive substances with diverse biological targets explains its efficacy on a broad range of human health conditions. The mechanisms of action of Pycnogenol^®^ were explored in several *in vitro*, *in vivo* and in clinical studies over the years (27). Randomized double-blind placebo-controlled (RDP) clinical trials help clarify and confirm some of the mechanisms of action of Pycnogenol^®^ at the molecular level. The protective effects of Pycnogenol^®^ on DNA, protein or lipid oxidation as well as its effects on blood antioxidant capacity were validated in several RDP studies on cardiovascular health, on cognitive function in older and young people, on menopause and on sports (9, 11–14, 56). Anti-inflammatory action of Pycnogenol^®^, like the reduction of inflammation mediators was shown in RDP trials on joint health as well as on respiratory health (40, 49). Pycnogenol^®^’s favorable impact on blood circulation was demonstrated in multiple ways, like the improvement of endothelial function or microcirculation in clinical RDP trials on cardiovascular health, chronic venous insufficiency, on skin and hair health, eye health and menopause (9, 11, 12, 20–22, 28–33, 43–47). Improved skin elasticity, skin moisture, joint flexibility and hair health illustrate the beneficial effects of Pycnogenol^®^ on the extracellular matrix, observed in RDP studies (40–44). The complementarity of these mechanisms justifies Pycnogenol^®^’s positive effects on its largely diversified areas of application.

Due to its unique production process and the sole usage of the plant species of *Pinus pinaster Ait. ssp. Atlantica* specific to the forest in Les Landes de Gascogne in France, the standardized pine bark extract registered as Pycnogenol^®^ stands unparalleled when compared with other pine bark extracts. The presented studies all used Pycnogenol^®^ supplementation and the obtained results cannot be extrapolated to other plant or pine bark extracts (5).

There are still gaps of knowledge on the exact mechanisms of action for some of the health applications of Pycnogenol^®^. In-depth investigations into the actions of the components and metabolites within cells are crucial. This not only enhances our comprehension of the effects of Pycnogenol^®^ in established fields but also opens avenues for exploring novel approaches to harness its potential benefits for human health. To unveil the full spectrum of bioactive metabolites and components in the human body, further investigations are warranted to pinpoint the most active compounds. Presently, only a limited number of bioactive metabolites of Pycnogenol^®^ have been identified. Utilizing advanced methods and expanded measurement capabilities in future research endeavors will undoubtedly reveal the comprehensive profile of active metabolites following the consumption of Pycnogenol^®^.

## Author contributions

FW: Conceptualization, Data curation, Writing – original draft, Writing – review & editing. PR: Supervision, Writing – review & editing.
